# Downregulation of miR-99b-5p and Upregulation of Nuclear mTOR Cooperatively Promotes the Tumor Aggressiveness and Drug Resistance in African American Prostate Cancer

**DOI:** 10.3390/ijms23179643

**Published:** 2022-08-25

**Authors:** Himali Gujrati, Siyoung Ha, Mohammad Waseem, Bi-Dar Wang

**Affiliations:** 1 Department of Pharmaceutical Sciences, University of Maryland Eastern Shore School of Pharmacy, Princess Anne, MD 21853, USA; 2 Hormone Related Cancers Program, University of Maryland Greenebaum Comprehensive Cancer Center, Baltimore, MD 21201, USA

**Keywords:** reciprocal miRNA-mRNA pairing, nuclear pmTOR, miR-99b-5p, precision prognostic biomarker

## Abstract

Mammalian target of rapamycin (mTOR) regulates various fundamental cellular events including cell proliferation, protein synthesis, metabolism, apoptosis, and autophagy. Tumor suppressive miR-99b-5p has been implicated in regulating PI3K/AKT/mTOR signaling in a variety of types of cancer. Our previous study suggested the reciprocal miR-99b-5p/*MTOR* (downregulated/upregulated) pairing as a key microRNA-mRNA regulatory component involved in the prostate cancer (PCa) disparities. In this study, we further validated the expression profiles of mTOR and miR-99b-5p in the PCa, colon, breast, and lung cancer specimens and cell lines. The immunohistochemistry (IHC), immunofluorescence, Western blot, and RT-qPCR assays have confirmed that mTOR is upregulated while miR-99b-5p is downregulated in different patient cohorts and a panel of cancer cell lines. Intriguingly, elevated nuclear mTOR expression was observed in African American PCa and other advanced cancers. Transfection of the miR-99b-5p mimic resulted in a significant reduction in nuclear mTOR and androgen receptor (AR), while a slight/moderate to no decrease in cytoplasmic mTOR and AR in PCa and other cancer cells, suggesting that miR-99b-5p inhibits mTOR and AR expression and their nuclear translocation. Moreover, overexpression of miR-99b-5p targets/inhibits AR-mTOR axis, subsequently initiating cell apoptosis and sensitizing docetaxel-induced cytotoxicity in various cancers. In conclusion, our data suggest that reciprocal miR-99b-5p/nuclear mTOR pairing may be a more precise diagnostic/prognostic biomarker for aggressive PCa, than miR-99b-5p/*MTOR* pairing or mTOR alone. Targeting the AR-mTOR axis using miR-99b-5p has also been suggested as a novel therapeutic strategy to induce apoptosis and overcome chemoresistance in aggressive PCa.

## 1. Introduction

Cancer has progressively been a major global health concern. In the United States, prostate cancer (PCa) is the most frequently diagnosed cancer (268,490 estimated new cases in 2022) and the second leading cause of cancer deaths (34,500 estimated deaths in 2022) among men [1]. A striking PCa disparity was observed, where African Americans (AAs) demonstrate a 1.7-fold higher incidence rate and a 2.3-fold higher mortality rate compared to their European American (EA) counterparts [1]. Besides the socioeconomic factors, accumulating genomic studies have suggested that genetic risk factors may also be involved in the PCa disparities [2,3,4,5]. MicroRNAs (miRNAs) are short non-coding RNAs regulating protein expression through degradation of target mRNAs or inhibition of protein translation. Emerging evidence has implicated miRNAs as potential biomarkers in cancers, including PCa [6,7,8,9]. A correlation-based approach, combining miRNA and mRNA (or protein) profiling data with mRNA target prediction to identify the miRNAs and mRNA targets with inverse regulatory correlation, has proved to be a more effective way to identify critical miRNA-mRNA interactions in cancers [10,11,12,13]. In our previous studies, we have applied an correlation-based, integrative genomic approach to define a novel panel of reciprocal miRNA-mRNA pairings (miR-34a-5p/*HIF1A*, miR-34a-5p/*PIK3CB*, miR-34a-5p/*IGFBP2*, miR-99b-5p/*MTOR*, miR-96-5p/*MAPKAPK2*, miR-133a/*MCL1*, miR-513c/*STAT1*, miR-96/*FOXO3A*, miR-145/*ITPR2*, and miR-34a/*PPP2R2A*) as critical regulatory components involved in AA PCa disparities [14,15]. These reciprocal miRNA–mRNA pairings (upregulated miRNA/downregulated mRNA target or downregulated miRNA/upregulated mRNA target, also indicated as up/down or down/up, in AA PCa vs. EA PCa) were demonstrated as key components differentially regulating the activation of ERBB, mTOR, and VEGF signaling pathways in AA PCa vs. EA PCa [14,15]. Mammalian target of rapamycin (mTOR) regulates a wide range of cellular events including cell proliferation, protein translation, metabolism, regeneration, autophagy, and apoptosis [16,17,18]. In the context of molecular mechanism, mTOR plays a central role coordinating ERBB (also known as EGFR/PI3K/AKT) and VEGF signaling in the ERBB/mTOR/VEGF axis, a signaling network frequently upregulated in PCa [19,20,21,22]. In this study, we particularly focused on the reciprocal pairing miR-99b-5p/*MTOR*, where miR-99b-5p is downregulated while *MTOR* is upregulated in AA PCa compared to EA PCa. To further explore the functional impacts of such miRNA-mRNA pairing in PCa disparities and to evaluate whether miR-99b-5p/*MTOR* (down/up) could potentially serve as a precision biomarker for aggressive PCa and other advanced cancers, a series of pathological, cellular/molecular biology and biochemical experiments were performed. First, immunohistochemistry (IHC) assays were conducted on tissue microarrays (TMAs) to examine the expression levels of mTOR in PCa specimens derived from EA and AA patients, and specimens derived from prostate, breast, colon, and lung cancer patients. Second, Western blot and RT-qPCR assays were performed to examine the expression levels of mTOR and miR-99b-5p, respectively, in both normal and cancerous cell lines derived from prostate, breast, colon and lung. Third, immunofluorescence assays were followed to investigate the expression levels and subcellular distributions of mTOR and its active form phosphorylated mTOR (pmTOR) in prostate, colon, breast, and lung cancer cell lines. Finally, an miR-99b-5p mimic was transfected to a panel of prostate, breast, colon, and lung cancer cell lines. The functional impacts of miR-99b-5p on regulating mTOR expression and cellular locations, cell apoptosis, and cytotoxic chemotherapy in these cancer cell lines were assessed by using immunofluorescence, Western blot, TUNEL, and apoptosis assays. Briefly, this study aimed to evaluate the potential of reciprocal miR-99b-5p/mTOR (down/up) as a novel diagnostic/prognostic biomarker, and to explore the plausible functional roles of miR-99b-5p in regulating cellular mTOR dynamics and the mTOR-mediating downstream signaling in PCa aggressiveness/progression. In summary, we demonstrated that mTOR is highly expressed while miR-99b-5p is downregulated in PCa and other cancers. In addition, elevated nuclear mTOR expression were observed in AA PCa and advanced cancer cells. These results suggest that miR-99b-5p/nuclear mTOR may serve as a potential diagnostic/prognostic biomarker for aggressive PCa and other cancers. Our functional assays have further implied that the miR-99b-5p-mediated AR/mTOR axis and nuclear mTOR expression/translocation may play critical functional roles for determining the PCa aggressiveness.

## 2. Results

### 2.1. Upregulation of mTOR in AA PCa Compared to EA PCa, and in PCa Compared to Normal

**Figure 1:**
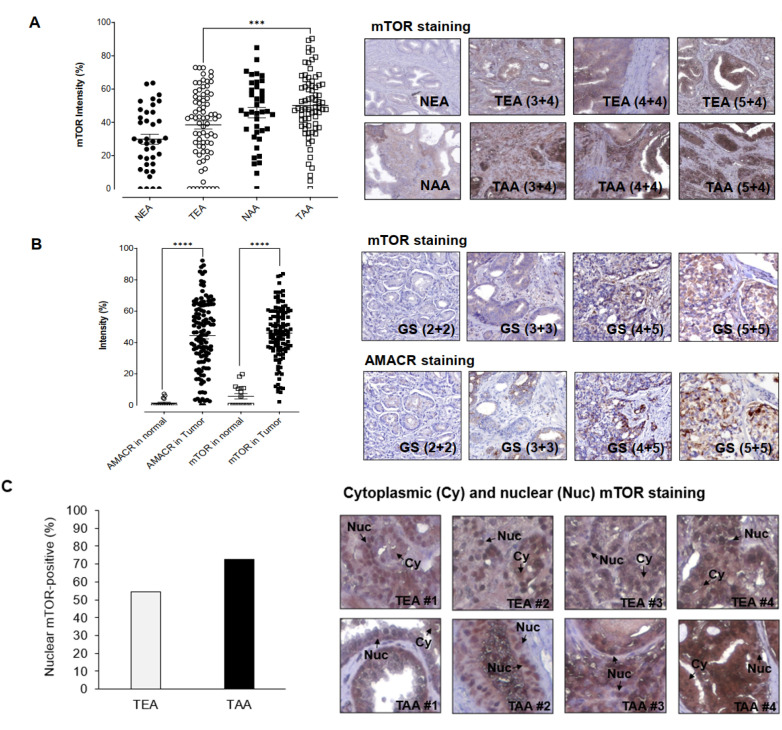
IHC staining assays for examining mTOR and AMACR protein levels in PCa patient specimens. (**A**) Quantification of IHC staining signals from mTOR in EA and AA PCa specimens (left panel) and representative IHC images from EA and AA PCa specimens (right panel). NEA: adjacent normal specimen derived from EA patient, TEA: tumorous EA specimen, NAA: adjacent normal specimen from AA PCa, TEA: tumorous AA specimen. Higher mTOR intensities were shown in AA PCa vs. EA PCa with comparable Gleason scores (3 + 4, 4 + 4, and 5 + 4). *** Significance (*p*-value < 0.001, comparing mTOR staining intensities in AA PCa vs. EA PCa specimens) was determined based on ANOVA with Tukey post-hoc test. (**B**) Quantification (left panel) and representative IHC images of mTOR and AMACR staining in PCa patient specimens (right panel). mTOR and AMACR staining intensities were measured in normal prostate tissues and PCa specimens in the TMAs. GS: Gleason Score. **** Significance (*p*-value < 0.0001, comparing AMACR or mTOR staining intensities in PCa vs. normal tissues) was determined based on paired *t*-test. (**C**) Higher frequency of nuclear mTOR signals was detected in AA PCa specimens (left panel). Representative IHC staining revealed both nuclear (Nuc) and cytoplasmic (Cy) mTOR expression in EA PCa (TEA) and AA PCa (TAA) specimens. Note that high-level nuclear (nearly exclusive) mTOR signals were detected in TAA #2 and #3 samples (right panel). Percentage of nuclear mTOR-positive TEA and TAA samples were calculated based on the equation of (number of nuclear mTOR-positive specimens/number of mTOR-positive specimens) × 100% in TEAs and TAAs, respectively.

Previously, our mRNA profiling, RT-qPCR, and Western blot results demonstrated that mTOR, targeted and inhibited by miR-99b-5p, is upregulated in AA PCa vs. EA PCa [14,15]. To further evaluate whether mTOR could serve as a potential diagnostic and/or prognostic biomarker for AA PCa (or PCa in general), IHC assays were performed to examine the mTOR expression levels in PCa specimens derived from two independent cohorts of PCa patients. Firstly, a formalin-fixed paraffin-embedded (FFPE) tissue microarray (TMA), containing cancerous specimens and adjacent normal tissues from 40–50 EA PCa and 40–50 AA PCa patients, and normal prostate tissues from 3 EA and 3 AA healthy individuals, was used to evaluate whether mTOR is differentially expressed between EA PCa and AA PCa. The IHC staining results have confirmed that AA PCa exhibited higher mTOR expression when compared to EA PCa. Quantification of mTOR intensities demonstrated that mTOR protein levels in tumorous AA PCa (TAA) specimens were significantly higher than tumorous EA PCa (TEA) specimens (Figure 1A, left panel). By comparing AA PCa and EA PCa samples with comparable Gleason scores (GS), significantly higher mTOR intensities were also observed in AA PCa vs. EA PCa specimens (Figure 1A, right panel). Notably, significantly higher mTOR expression was detected even in noncancerous NAA samples vs. NEA samples (Figure 1A, left and right panels), suggesting that mTOR may be a predisposing risk factor in AAs (who have higher chance to develop aggressive types of PCa).

Secondly, two TMAs containing 16 normal prostate tissues, 7 hyperplasia specimens, and 169 PCa specimens (from US Biomax Inc., Derwood, MD, USA) were used to examine the mTOR and α-methylacyl CoA racemase (AMACR) expression levels, respectively. Several reports indicated that AMACR has emerged as an PCa biomarker [23,24,25,26], and AMACR expression level has also been associated with PCa progression and prognosis [27,28]. As shown in Figure 1B, AMACR and mTOR expression levels were significantly higher in PCa specimens than in normal tissues. Overall, comparable expression levels of AMACR and mTOR were observed in PCa samples (Figure 1B, left panel). Moreover, mTOR intensities seems to be positively correlated to the AMACR levels in the PCa samples derived from the same existing patients (Figure 1B, right panel). These results suggest that mTOR, with similar AMACR expression profile in PCa specimens, may potentially serve as a potential biomarker for diagnosis and/or prognosis in PCa. The IHC assays have further revealed that mTOR was expressed in both cytoplasmic and nuclear fractions of the PCa samples (Figure 1C, left and right panels). Notably, a higher percentage of AA PCa specimens (72.6%, 53 out of 73 AA PCa specimens expressed nuclear mTOR) demonstrated nuclear mTOR signals, when compared to the EA PCa specimens (54.3%, 38 out of 70 EA PCa samples expressed nuclear mTOR) (Figure 1C, left panel). Considering the aggressive nature of AA PCa (i.e., higher recurrence and mortality rates), it raised an interesting question as to whether the high nuclear mTOR level (a more oncogenic form of mTOR) functionally contributes to the AA PCa aggressiveness.

### 2.2. Differential Subcellular Distributions of mTOR and pmTOR in AA PCa and EA PCa

**Figure 2:**
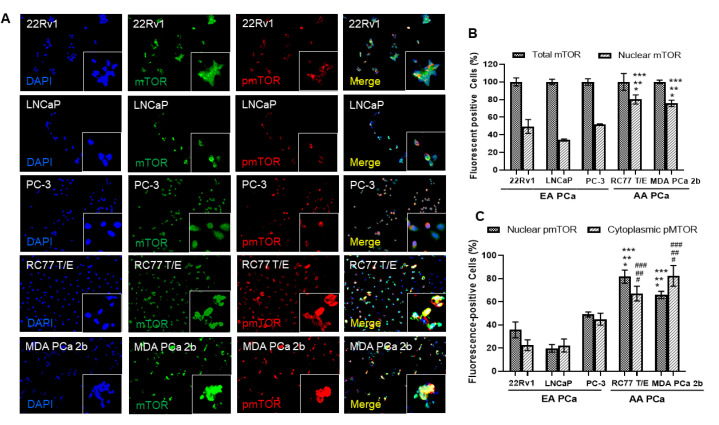
Immunofluorescence staining demonstrated higher expression levels of mTOR, nuclear mTOR, and nuclear pmTOR in AA PCa when compared to EA PCa cells. (**A**) Immunofluorescence showing mTOR (green fluorescence) and pmTOR (red fluorescence) signals in EA PCa cell lines (22Rv1, LNCaP and PC-3), and AA PCa cell lines (RC77 T/E and MDA PCa 2b). Nuclei were visualized by counterstaining with DAPI (blue fluorescence). Merged images were achieved by overlaying DAPI, mTOR and pmTOR signals to identify colocalization (yellow) of mTOR and pmTOR in nuclei. Fluorescent image capturing and analysis were performed using CellScans software V1.18. (**B**) Total mTOR and nuclear mTOR signals in EA and AA PCa cell lines. Fluorescence mTOR- or pmTOR-positive cells (%) were determined based on (number of mTOR or pmTOR-positive cells/number of DAPI-positive cells) × 100%. Significance was determined based on ANOVA with Tukey’s post-hoc test (* *p*-value < 0.001 in 22Rv1 vs. RC77 T/E or vs. MDA PCa 2b, ** *p*-value < 0.001 in LNCaP vs. RC77 T/E or vs. MDA PCa 2b, and *** *p*-value < 0.001 in PC-3 vs. RC77 T/E or vs. MDA PCa 2b). (**C**) Distribution of cytoplasmic and nuclear pmTOR in EA and AA PCa cell lines. Significant difference of nuclear mTOR (* *p*-value < 0.001 in 22Rv1 vs. RC77 T/E or vs. MDA PCa 2b, ** *p*-value < 0.001 in LNCaP vs. RC77 T/E or vs. MDA PCa 2b, and *** *p*-value < 0.001 in PC-3 vs. RC77 T/E or vs. MDA PCa 2b) and nuclear pmTOR (^#^ *p*-value < 0.001 in 22Rv1 vs. RC77 T/E or vs. MDA PCa 2b, ^##^ *p*-value < 0.001 in LNCaP vs. RC77 T/E or vs. MDA PCa 2b, and ^###^ *p*-value < 0.001 in PC-3 vs. RC77 T/E or vs. MDA PCa 2b) were shown in AA PCa vs. EA PCa. The statistics were determined based on ANOVA with Tukey’s post-hoc test, and each value was represented as mean ± SEM (*n* = 6).

To further explore the potential functional roles of cytoplasmic and nuclear mTOR in AA PCa, immunofluorescence assays were conducted in three EA PCa (22Rv1, LNCaP, and PC-3) and two AA PCa (RC77 T/E, and MDA PCa 2b) cell lines. The PCa 22Rv1 represents an androgen-independent EA PCa cell line model, while LNCaP and PC-3 are metastatic EA PCa cell lines derived from lymph node and bone metastasis. RC77 T/E was used as a primary AA PCa cell model, while MDA PCa 2b was used as a metastatic AA PCa cell line derived from bone metastasis. The immunofluorescence assays have revealed higher levels of cytoplasmic mTOR vs. nuclear mTOR in 22Rv1, LNCaP, and PC-3 cells, whereas nearly equal distribution of cytoplasmic and nuclear mTOR were observed in RC77 T/E and MDA PCa 2b (green fluorescent mTOR signals and DAPI signals in Figure 2A). Specifically, ~80% of AA PCa (RC77 T/E and MDA PCa 2b) cells expressed nuclear mTOR, but only 30–45% of EA PCa (22Rv1, LNCaP, and PC-3) cells expressed nuclear mTOR (Figure 2B). In addition, significantly higher pmTOR (the active form of mTOR) levels were shown in both cytoplasmic and nuclear fractions of AA PCa cell lines than EA PCa cell lines (Figure 2A,C). Notably, metastatic AA PCa (MDA PCa 2b) cells exhibited the highest ratio of nuclear pmTOR/total mTOR (~80%, calculated based on the number of nuclear pmTOR-positive cells/the number of mTOR-positive cells × 100%) among all PCa cell lines (red fluorescence, DAPI and merged signals in Figure 2A, and quantification data in Figure 2C). The differential distributions of cytoplasmic/nuclear mTOR and pmTOR between AA PCa and EA PCa suggest that more oncogenic events (resulted from higher level of nuclear mTOR-mediated transcriptional activation of downstream/metabolic genes in chromatin) may occur in AA PCa vs. EA PCa. This raises an interesting question whether and how the elevated nuclear mTOR and pmTOR protein levels contributes to the more aggressive phenotypes of AA PCa.

### 2.3. Transfection of miR-99b-5p Inhibits the Expression Levels and Nuclear Translocation of Total mTOR and Nuclear pmTOR in PCa Cells

**Figure 3:**
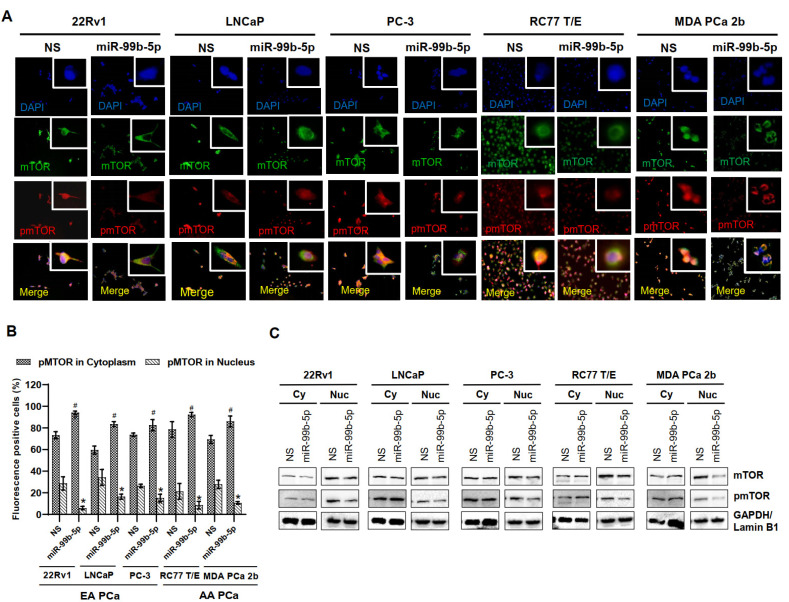
Transfection of miR-99b-5p mimic attenuates mTOR and pmTOR expressions and blocks the translocation of pmTOR to the nuclei. (**A**) Immunofluorescence showing cytoplasmic and nuclear localizations of mTOR (green fluorescence) and pmTOR (red fluorescence) in EA PCa cell lines (22Rv1, LNCaP and PC-3) and AA PCa cell lines (RC77 T/E and MDA PCa 2b) transfected with NS or miR-99b-5p mimic. The nuclei were visualized by counterstaining with DAPI. (**B**) Quantification analysis of cytoplasmic and nuclear distribution in EA and AA PCa cells. Significant increase in cytoplasmic pmTOR (^#^ *p*-value < 0.01 using ANOVA with Tukey’s post-hoc test) and a significant decrease in nuclear pmTOR (* *p*-value using ANOVA with Tukey’s post-hoc test) was observed in the miR-99b-5p mimic vs. NS transfected cells. Each value was represented as mean ± SEM (*n* = 6). (**C**) Western blot analysis of mTOR and pmTOR in cytoplasmic and nuclear fractions of EA and AA PCa cell lines transfected with NS or the miR-99b-5p mimic. The representative images shown here were selected from 3–4 independent Western blot results. GAPDH and Lamin B1 were used as endogenous controls for cytoplasmic (Cy) and nuclear (Nu) proteins, respectively.

To assess our hypothesis that the AA PCa aggressiveness may be due to the overall upregulation of nuclear mTOR and pmTOR, nonsense/scrambled RNA (NS) or miR-99b-5p mimic was transfected to the EA PCa and AA PCa cell lines then followed by examining the subcellular distributions/expression levels of mTOR and pmTOR by using immunofluorescence assays and Western blot analysis. A generalized reduction in total mTOR (green fluorescence) and pmTOR (red fluorescence) signals were observed across all EA and AA PCa cell lines upon miR-99b-5p transfections (Figure 3A). Intriguingly, higher ratio of cytoplasmic pmTOR and lower ratio of nuclear pmTOR signals were observed in PCa cells transfected with miR-99b-5p mimic vs. NS control. This phenomenon is particularly evident in the AA PCa cell lines RC77 T/E and MDA PCa 2b, where the high nuclear pmTOR/mTOR signals in NS transfected cells but exclusively cytoplasmic pmTOR/mTOR signals in miR-99b-5p transfected cells were observed (merged images, RC77 T/E and MDA PCa 2b transfected with NS vs. miR-99b-5p mimic, Figure 3A). Quantification of both cytoplasmic and nuclear pmTOR signals further confirmed that the cytoplasmic pmTOR signals were increased and the nuclear pmTOR signals were reduced significantly upon miR-99b-5p overexpression in all EA and AA PCa cell lines (Figure 3B). Western blot assays were used to further verify the protein dynamics/distribution of mTOR and pmTOR in the cytoplasmic and nuclear fractions of EA and AA PCa cells. The EA and AA PCa cell lines were transfected with NS or miR-99b-5p mimic for 48 h, the cytoplasmic and nuclear proteins were prepared and subjected to the Western blot analysis. Consistent with the immunofluorescence results, the Western blot assays have again demonstrated a moderate to significant reduction in mTOR and pmTOR in nuclear fractions from all EA and AA PCa cell lines transfected with miR-99b-5p mimic (Figure 3C, and Supplementary Figure S2). It is particularly evident from the significant decrease in nuclear mTOR and pmTOR in the metastatic AA PCa cell line MDA PCa 2b (Figure 3C, and Supplementary Figure S2). In contrast, miR-99b-5p transfection caused a slight to no reduction in cytoplasmic mTOR in all the PCa cell lines (Figure 3C, and Supplementary Figure S2). Taken together, these results strongly suggest that miR-99b-5p overexpression inhibits mTOR expression and may also blocks the translocation of mTOR (as well as pmTOR) from cytoplasm to nucleus in PCa.

### 2.4. Upregulation of mTOR and Downregulation of miR-99b-5p in Colon, Breast and Lung Cancer Specimens and Cell Lines

**Figure 4:**
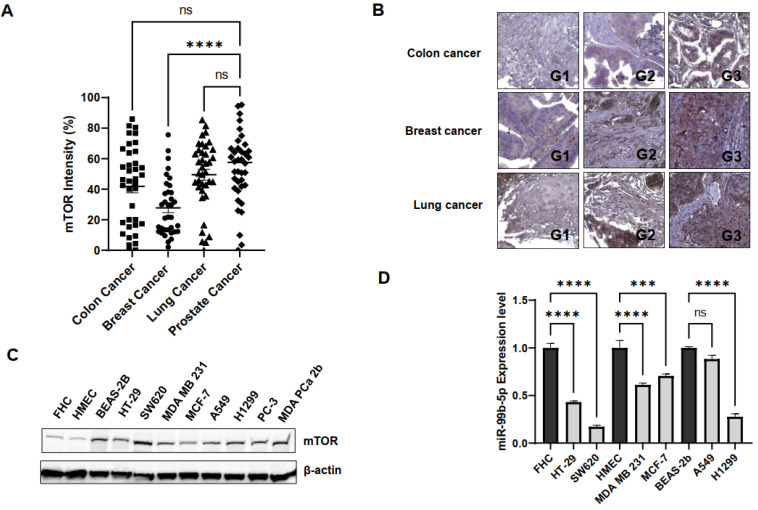
mTOR and miR-99b-5p expression profiles in colon, breast, and lung cancer cell models. (**A**) Quantification of mTOR intensities in colon, breast, lung, and prostate cancer specimens using IHC staining assay. IHC staining assay was applied to examine mTOR protein levels in various solid tumor patient specimens on a TMA slide. Significantly different mTOR intensities were identified in PCa vs. breast cancer specimens (**** *p*-value < 0.0001, based on ANOVA with Dunnett’s post-hoc test). No significant difference (ns) in mTOR intensities was found in colon cancer vs. PCa, or lung cancer vs. PCa specimens. (**B**) Representative IHC staining images of the mTOR protein in colon, breast, and lung cancer specimens. Apparently, the expression levels of mTOR were gradually increased from low- to high-grade cancer samples. G1: grade 1 tumor, G2: grade 2 tumor, and G3: grade 3 tumor. (**C**) Western blot analysis of mTOR protein levels in a panel of normal and cancer cell lines. Representative Western blot analysis of mTOR protein levels from total protein extracts of colon cancer (HT-29, SW620), breast cancer (MDA MB 231, MCF-7), lung cancer (A549, H1299), and PCa (PC-3, MDA PCa 2b) cell lines and control cell lines (FHC, HMEC and BEAS-2B from normal colon, breast and lung tissues, respectively). (**D**) RT-qPCR assays showed downregulation of miR-99b-5p in colon, breast and lung cancer cell lines, compared to their normal controls. RNA samples isolated from FHC, HMEC, HT-29, SW620, MDA MB 231, MCF-7, BEAS-2B, A549, and H1299 were subjected to RT-qPCR assays of miR-99b-5p. Significantly different miR-99b-5p expression levels (**** *p*-value < 0.0001 and *** *p*-value < 0.001, based on ANOVA with Tukey’s post-hoc test) were shown in cancer cell lines vs. normal controls (except A549 vs. BEAS-2B). ns: not significant. Each value was represented as mean ± SEM, obtained from three independent cDNA samples with duplicate or triplicate qPCR reactions. ns: not significant.

To further evaluate whether the reciprocal mTOR/miR-99b-5p pairing can also serve as a potential biomarker for solid tumors in general, IHC, Western blot, and RT-qPCR assays were performed to examine the expression levels of the mTOR protein and miR-99b-5p in colon, breast, and lung cancers. The IHC assay on a TMA containing multiple cancer specimens has shown that the mTOR protein is overexpressed in colon, breast, and lung cancer patient specimens (Figure 4A). Statistically, the colon and lung cancer specimens expressed comparable mTOR levels when compared to PCa samples in the TMA, while breast cancer specimens expressed lower levels of mTOR protein than the PCa specimens (Figure 4A). Furthermore, IHC staining results have revealed that increased mTOR levels were detected in high-grade/advanced colon, breast, and lung cancer specimens. For instance, the highest mTOR intensities were detected in grade 3 cancers, while moderate and low mTOR intensities were detected in grade 2 and 1 patient specimens, respectively (Figure 4B). The positive correlation between mTOR staining intensities and tumor grades suggests that the mTOR expression profile may be a potential index/biomarker for evaluating cancer aggressiveness. Interestingly, the survival curves generated from RNA-sequencing (RNA-seq) database of the Cancer Genome Atlas (TCGA) also demonstrated that a higher mTOR expression level is correlated with an overall poorer survival in colon, breast, lung, and pancreatic cancer patients (Supplementary Figure S1). These results suggest that the mTOR expression profile may potentially serve as a precision prognostic biomarker (i.e., correlated to cancer grades/aggressiveness, clinical outcomes, etc.) for PCa and other solid tumors.

To further validate mTOR and miR-99b-5p expression levels in the in vitro cancer cell line models, Western blot analysis and RT-qPCR assays were conducted in a panel of colon, breast, lung, and prostate cancer cell lines. First, the Western blot analysis has shown that mTOR is upregulated in colon cancer (HT-29 and SW620) vs. normal colon (FHC) cells, in breast cancer (MDA MB 231, MCF-7) vs. normal breast (HMEC) cells, in lung cancer (A549 and H1299) (Figure 4C). Note that all the cancer cell lines expressed similar mTOR levels when compared to the mTOR-expressing PCa cell lines (PC-3 and MDa PCa 2b). The only exception is that normal lung cell line BEAS-2B also expresses mTOR with comparable level as other cancer cells (Figure 4C).

Next, the expression levels of miR-99b-5p (a negative regulator of mTOR) were examined in the same panel of the cancerous and normal cell lines used in Western blot analysis. RT-qPCR results have confirmed that miR-99b-5p was significantly downregulated in colon cancer (HT-29 and SW620) vs. normal colon (FHC) cells, in breast cancer (MDA MB 231, MCF-7) vs. normal breast (HMEC) cells, and in lung cancer (H1299) vs. normal lung (BEAS-2B) cells (Figure 4D). The negative correlation of mTOR and miR-99b-5p expression levels in colon, breast, and lung cancer cells indicated that the regulatory effects of the reciprocal pairing miR-99b-5p/mTOR (down/up) may play a crucial role in advanced colon, breast, and lung cancers, similar to what we observed in the AA PCa [15].

### 2.5. Overexpression of miR-99b-5p Reduces Expression of mTOR and pmTOR, Inhibits the Nuclear Translocation of pmTOR and Induces Cell Apoptosis in Various Cancer Cells

**Figure 5:**
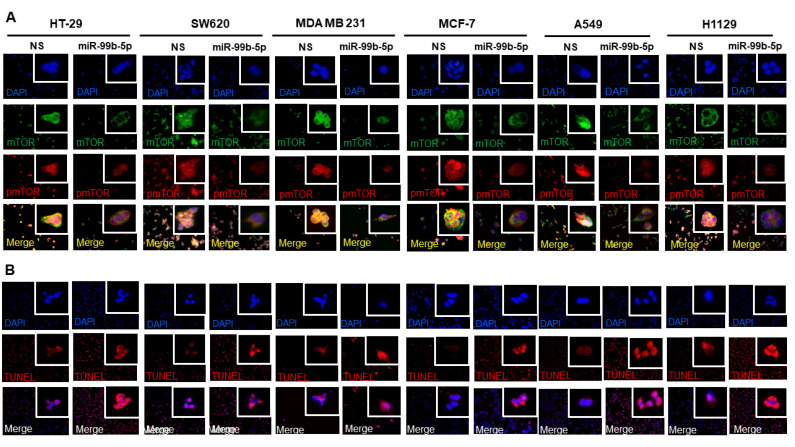
Overexpression of miR-99b-5p changes the subcellular distribution of mTOR and pmTOR and initiates cell apoptosis. (**A**) Immunofluorescence assays were used to visualize the subcellular localization of mTOR (green fluorescence) and pmTOR (green fluorescence) signals in cancer cell lines (HT-29, SW620, MDA MB 231, MCF-7, A549, and H1299) transfected with NS or miR-99b-5p mimic. Nuclei were visualized by counterstaining with DAPI. (**B**) TUNEL assays were used to visualize the DNA damages created during apoptotic events in the cancer cell lines upon miR-99b-5p and NS transfections. Apoptotic events were detected based on the DNA damages (visualized as red fluorescent spot signals, TUNEL panel) in the nuclei (blue, DAPI panel). The red/purple signals shown by overlaying DAPI and TUNEL signals (Merge panel) indicated apoptotic activities (DNA damages occurring in the nuclei) in the cancer cells.

Various cancer cell lines (HT-29, SW620, MDA MB 231, MCF-7, A549, and H1299) were used as solid tumor cell models to investigate the functional impacts of miR-99b-5p overexpression in mTOR expression and subcellular distribution. Similar to the effects of miR-99b-5p in PCa cells, all the six cancer cell lines showed a generalized reduction in mTOR (green fluorescence) and pmTOR (red fluorescence) levels in miR-99b-5p transfected vs. NS transfected cells (Figure 5A). Further analyzing the subcellular distribution of mTOR and pmTOR has revealed elevated cytoplasmic mTOR/nuclear mTOR ratios (as well as cytoplasmic pmTOR/nuclear pmTOR ratios) in miR-99b-5p mimic vs. NS transfected cancer cells (Figure 5A). Specifically, higher levels of nuclear mTOR and/or nuclear pmTOR signals were observed in the NS-transfected cells (green, red fluorescence, and yellow merge in nuclei, Figure 5A). In contrast, the mTOR and pmTOR were mostly expressed in the cytoplasm of the miR-99b-5p mimic transfected cells (green and red in cytoplasm, while slight to no green/red colocalization in nuclei merge, miR-99b-5p panel in Figure 5A). These results suggest that miR-99b-5p targets/inhibits mTOR in cancer cells, subsequently blocking mTOR translocation from cytoplasm to nucleus. Furthermore, TUNEL assays were conducted to assess whether the miR-99b-5p overexpression can induce the cell apoptosis. As shown in Figure 5B, TUNEL assay results have demonstrated enhanced DNA breakages (i.e., increased red fluorescent signals in nuclei) in the miR-99b-5p mimic vs. NS transfected cancer cells, implicating that miR-99b-5p initiates/promoting cell apoptosis in the cancer cells.

### 2.6. Transfection of miR-99b-5p Mimic Inhibits AR-Mediated mTOR Translocation and Promotes the Docetaxel-Induced Cytotoxicity in Various Cancers

**Figure 6:**
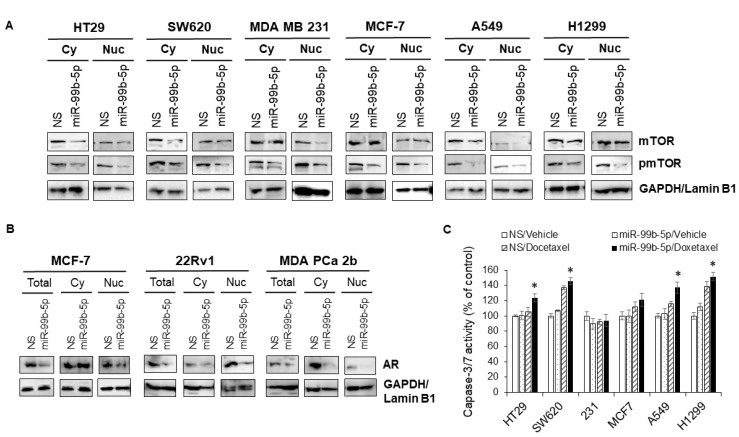
Overexpression of miR-99b-5p inhibits mTOR and pmTOR expression and nuclear translocation, and sensitizes the docetaxel-induced cytotoxicity in cancer cells. Western blot analyses were used to examine: (**A**) mTOR and pmTOR protein levels in cytoplasmic and nuclear fractions. GAPDH and Lamin B1 were used as endogenous controls for cytoplasmic (Cy) and nuclear (Nu) proteins, respectively. (**B**) AR levels in total cell lysates (Total), cytoplasm (Cy), and nuclei (Nu) from the cancer cells (MCF-7, 22Rv1, and MDA PCa 2b) transfected with NS or miR-99b-5p mimic. The representative images were selected from 3–4 independent Western blot results. GAPDH was used as endogenous control for total and cytoplasmic proteins, and Lamin B1 was used as endogenous protein control for the nuclear proteins. (**C**) Apoptosis assays were performed in the various cancer cell lines transfected with NS or miR-99b-5p mimic in the absence or presence of 11nM docetaxel. Significantly different apoptosis capacity (* *p*-value < 0.05, in miR-99b-5p transfected cells treated with docetaxel vs. vehicle) were determined based on ANOVA with Tukey’s post-hoc test. Each value was represented as mean ± SD (*n* = 3–4); 231: MDA MB 231.

To explore the molecular mechanism of miR-99b-5p-mediated mTOR expression and cytoplasmic/nuclear mTOR dynamics, Western blot analyses were performed after the cancer cells were transfected with NS or the miR-99b-5p mimic in the absence or presence of docetaxel. First, Western blot analysis has again confirmed that miR-99b-5p negatively regulates mTOR protein expression in these cancer cells, evident from a generalized reduction in total mTOR (combined protein levels of cytoplasmic and nuclear mTOR) and a significant decrease in nuclear mTOR (and nuclear pmTOR) expression in miR-99b-5p vs. NS transfected cells (Figure 6A, and Supplementary Figure S3). In contrast, slight to no decrease in cytoplasmic mTOR was observed in cancer cells upon miR-99b-5p transfection (Figure 6A, and Supplementary Figure S3). These results, again, suggest that miR-99b-5p targets/inhibits mTOR and mediates (directly or indirectly) mTOR translocation to the nucleus.

Androgen receptor (AR) plays a central role for PCa pathogenesis.

Previous studies have revealed that AR activation mediates the mTOR signaling, and the formation of AR–mTOR complex is required for the translocation of mTOR from cytoplasm to nucleus. However, it remains unknown how miR-99b-5p participates at the AR–mTOR axis and regulates the mTOR translocation to nucleus. To explore the possible molecular mechanism underlying the miR-99b-5p-mediated cytoplasmic/nuclear mTOR dynamics and the downstream effects on cell survival/apoptosis, Western blot analyses were performed to examine the protein levels of AR in total, cytoplasmic, and nuclear protein fractions. Breast cancer (MCF-7) and PCa (22Rv1 and MDA PCa 2b) cell lines that express considerable levels of AR were used as in vitro cell line models to study the miR-99b-5p involvement in AR-mTOR axis. Similar to the miR-99b-5p effect on mTOR expression/location, the AR expression levels were significantly reduced in miR-99b-5p mimic vs. NS transfected cancer cells (total lysates, Figure 6B). Moreover, nuclear AR protein levels were significantly decreased in MCF-7, 22Rv1, and MDA PCa 2b cells. In contrast, miR-99b-5p overexpression caused no change of cytoplasmic AR in MCF-7 cells, and slight to moderate decrease in cytoplasmic AR in 22Rv1 and MDA PCa 2b cells (Figure 6B). Taken together, our data suggest that miR-99b-5p negatively regulates mTOR and AR, subsequently inhibiting the translocation of AR/mTOR complex from cytoplasm to nucleus.

Next, we further investigated whether the miR-99b-5p can target/inhibit the AR/mTOR axis and promote cell apoptosis and/or enhance the docetaxel-induced cytotoxicity in colon, breast, and lung cancers. The caspase 3/7 activity-based apoptosis assays have revealed that miR-99b-5p as a single agent was not sufficient to initiate significant apoptosis in the tested cancer cells. However, miR-99b-5p overexpression induced moderate to significant cell apoptosis in the presence of chemotherapeutic agent docetaxel in all cancer cell lines, except MDA MB 231 (Figure 6C). These results, similar to our previous observation in PCa [15], again suggest that miR-99b-5p targets/inhibits mTOR signaling and consequently sensitizes the docetaxel-induced cytotoxicity in the advanced cancer cells.

## 3. Discussion

In the current study, we investigated whether miR-99b-5p/*MTOR* can serve as a precision diagnostic and/or prognostic biomarker in PCa and other solid tumors including colon, breast and lung cancers. Our IHC, Western blot, and RT-qPCR results have confirmed an overall upregulation of mTOR and downregulation of miR-99b-5p in independent cohorts of patient samples and a panel of cell lines derived from prostate, colon, breast and lung cancers. In vitro functional assays further demonstrated that miR-99b-5p targets AR–mTOR signaling, resulting in inhibiting AR and mTOR expression, blocking nuclear translocation of mTOR, enhancing cell apoptosis, and sensitizing docetaxel-induced cytotoxicity in PCa cells. Similar functional effects of miR-99b-5p on AR/mTOR inhibition, mTOR localization, and apoptosis induction were also shown in colon, breast, and lung cancer cell models.

Previous studies have shown that the tumor suppressive miR-99b-5p is downregulated in various cancers including PCa [15,29,30,31,32]. In contrast, mTOR is upregulated in a variety of cancer types [15,16,33]. Our recent publication has further suggested a potential clinical application of utilizing reciprocal miR-99b-5p/mTOR (down/up) pairing as a diagnostic/prognostic biomarker in AA PCa [15]. In this study, we further confirmed that mTOR is upregulated in AA PCa vs. EA PCa and PCa vs. normal (Figure 1). Furthermore, an inverse correlation between miR-99b-5p and mTOR expression levels was confirmed in other solid tumors including colon, breast, and lung cancers (Figure 4). Although data analysis from TCGA RNA-seq data has demonstrated an overall lower survival rate in cancers expressing high-level vs. low-level *MTOR* transcript, the differences were not statistically significant (Supplementary Figure S1). Further survival data analysis by extracting RNA-seq data from patients expressing high-level *MTOR* but low-level miR-99b-5p may further validate whether reciprocal miR-99b-5p/*MTOR* (down/up) pairing is a better prognostic biomarker than mTOR alone.

Another interesting observation is that AA PCa exhibits a higher level of nuclear mTOR than EA PCa. This raises a challenging question of whether the elevated nuclear mTOR expression contributes to the more aggressive properties observed in AA PCa. Emerging studies have indicated the unique functions of nuclear mTOR in cancers including PCa. Although the mTOR protein is mainly localized in cytoplasm, nuclear mTOR and its oncogenic impacts have been implicated in several tumors, including gastric cancer, endometrial cancer, thyroid cancer, PCa and multiple myeloma [34,35,36,37,38,39]. For instance, higher nuclear mTOR expression has been associated with poor prognosis in endometrial, thyroid, and prostate cancers [34,35,37].

Overexpression of miR-99b-5p has been reported to negatively regulate the protein expression of mTOR, AR and prostate specific antigen (PSA), consequently inhibiting the cell proliferation, migration, inducing autophagy, promoting apoptosis, and sensitizing the docetaxel-induced cytotoxicity in PCa [32]. AR has also been implicated in the coordination of protein complex diversity and subcellular localization of mTOR, including regulating the translocation of mTOR to the nucleus and the mTOR-mediated gene networks [34,38]. It is reported that AR activation leads to the upregulation of mTOR signaling, and enhances mTOR translocation from cytoplasm to nucleus. ChIP-seq and ChIP-qPCR results further revealed that AR and mTOR colocalize at the same genomic loci, and the mTOR-chromatin binding is driven in an AR-dependent manner in PCa cells. Mechanistically, the nuclear mTOR/AR signaling axis mediates the metabolic reprogramming in PCa [34]. Consistent with the previous studies, our data have demonstrated that miR-99b-5p negatively regulates mTOR and AR expression, initiates cell apoptosis, and promotes the docetaxel-induced cytotoxicity in PCa and other solid tumor cells. Moreover, our immunofluorescence and Western blot analysis further revealed that miR-99b-5p inhibits nuclear translocation of mTOR. This is particularly evident form the significant reduction in nuclear mTOR, pmTOR and AR, but slight/moderate to no reduction in cytoplasmic mTOR and AR in PCa and other cancer cells upon miR-99b-5p mimic transfection (Figure 3, Figure 5 and Figure 6). Here, we proposed a molecular model that miR-99b-5p negatively regulates the expression level of AR and mTOR, thereby decreasing the overall level of AR/mTOR complex for nuclear translocation. In fact, miR-99b-5p also targets/inhibits SMARCD1 [40], a cofactor of active AR [41], which may also involve in the miR-99b-5p-mediated nuclear translocation of mTOR. To the best of our knowledge, this is the first report to propose a functional mechanism of miR-99b-5p/AR/mTOR signaling axis in regulating PCa aggressiveness and progression. Previous study has also demonstrated that *IGF1R* (encoding insulin-like growth factor 1 receptor, IGF-1R), an upstream gene of AKT/mTOR signaling, is a direct target of miR-99b-5p in gastric cancer [29]. Although no differential *IGF1R* expression in AA vs. EA PCa was identified from our mRNA profiling data [14], the downregulation of miR-99b-5p may additionally stimulate the IGF-1F-mediated AKT/mTOR signaling in PCa progression, regardless of the ethnicities.

In conclusion, our study provides a molecular insight into how miR-99b-5p/AR/mTOR axis regulates the PCa aggressiveness and progression. Instead of considering total mTOR as an oncogenic indicator, our results suggest that reciprocal miR-99b-5p/“nuclear” mTOR pairing (down/up) pairing may serve as a potential precision diagnostic and prognostic biomarker for PCa. Further developing a dual staining protocol for miR-99b-5p and nuclear mTOR (or nuclear pmTOR) using RNAScope/IHC technology may facilitate the development of a precision diagnostic/prognostic biomarker for aggressive PCa. Lastly, a deeper understanding of the molecular mechanism underlying miR-99b-5p-mediated AR/mTOR signaling axis and AR/mTOR cytoplasmic/nuclear dynamics may pave a new path for developing novel therapeutics to treat the aggressive PCa.

## 4. Materials and Methods

### 4.1. Cell Culture

The human cell lines used in the study included: 22Rv1, LNCaP, PC-3, RC77 T/E and MDA PCa 2b (PCa cell lines), MDA MB 231 and MCF-7 (breast cancer cell lines), HT-29 and SW620 (colon cancer cell lines), A549 and H1299 (lung cancer cell lines). RWPE-1, HMEC, FHC, and BEAS-2B were used as normal control cell lines for prostate, breast, colon, and lung, respectively. Note that 22Rv1 is a castration-resistant EA PCa cell line, while LNCaP and PC-3 were derived from lymph node and bone metastasis of EA PCa patients, respectively. RC77 T/E was derived from a primary AA PCa patient, and MDA PCa 2b was derived from bone metastasis of an AA PCa patient. The cell lines described were used as in vitro cell models to evaluate the functional roles of miR-99b-5p/*MTOR* pairing in the EA PCa, AA PCa and solid tumors in general. The cells were grown in specific cell culture media described as follows: 22Rv1, LNCaP, MCF-7 and H1299 were cultured in RPMI-1640 with 10% fetal bovine serum (FBS), PC-3, A549 and FHC were cultured in DMEM with 10% FBS, RC77 T/E was cultured in Keratinocyte SFM with human recombinant epidermal growth factor (EGF) and bovine pituitary extract (BPE), MDA PCa 2b were cultured in BRFF-HPC1 with 20% FBS, HT-29 was cultured in McCory’s with 10% FBS, SW620 and MDA MB 231 were cultured in L-15 with 10% FBS, HMEC was cultured in mammary epithelial cell basal medium with supplements, and BEAS-2B was cultured in BEBM base medium with BEBM supplement kit. Cells were maintained at 37 °C in a 5% CO_2_ incubator.

### 4.2. Transfection of miR-99b-5p Mimic and Nonsense/Scrambled RNA in Cell Line Models

The 22Rv1, LNCaP, PC-3, MDA PCa 2b, MCF7, H1299, A549, HT-29, SW620, MDA MB-231, HMEC and BEAS-2B cells were seeded at a density of 3 × 10^5^ cells/well in 6-well plates. RC77 T/E cells were seeded at a density of 5 × 10^5^ cells/well in 6-well plates. The cells were grown for 24 h and then transfected with nonsense/scrambled RNA (NS) and miR-99b-5p mimic (Ambion, Austin, TX, USA) using DharmaFECT4 transfection reagent (Dharmacon, Lafayette, CO, USA). After 24 h, fresh media were applied to replace the transfection reagent-containing media, then the cells were incubated for an additional 24 h.

### 4.3. Tissue Microarrays (TMAs)

Three types of TMAs were used in this study. First, a TMA containing EA and AA PCa samples, adjacent normal prostate tissues from 40–50 EA and 40–50 AA PCa patients, as well as normal prostate tissues from 3 healthy EA and 3 AA healthy individuals was used to evaluate the mTOR expression levels using IHC assay. This type of TMA was designed and prepared by the Department of Pathology at University of Maryland Baltimore (UMB). Second, TMAs containing normal prostate tissue and PCa specimens were purchased from US Biomax Inc. (catalog# PR208a, Derwood, MD, USA). The PCa TMAs were used to examine the mTOR and AMACR expression levels in the PCa patient samples and adjacent normal tissues included on the TMAs. Third, a TMA containing tumor samples derived from patients diagnosed with breast cancer, colon cancer, and lung cancer (catalog# BC000119b, US Biomax, Derwood, MD, USA) was used for examining the expression levels of mTOR.

### 4.4. Immunohistochemistry (IHC) Assays

Slides containing serial sections were deparaffinized in xylene (2 × 5 min), followed by immersion in xylene/alcohol solution (xylene:ethanol = 1:1) and slowly rehydrated through graded alcohols (100%, 95%, 70% and 50% of alcohol, respectively) to distilled water. EnVision FLEX target retrieval solution from Agilent technologies (Carpinteria, CA, USA) was purchased and diluted according to the manufacturer’s protocol. Antigen retrieval was performed in microwave for 25 min (with full power for 5 min, and 20% power for an additional 20 min), followed by cooling down sections for 30 min at room temperature. The sections were then rinsed with running cold water for 10 min. Peroxidase block was added dropwise and incubated for 30 min at room temperature. The slides were then washed with 1 × PBS twice for 5 min followed by adding blocking buffer (2.5% BSA in 1 × PBS) and incubating for 30 min at room temperature. After discarding blocking buffer, tissue sections were incubated with the primary antibody (1:100–1:200 dilutions in 2.5% BSA/1 × PBS) at 4 °C overnight. On the following day, sections were washed with 1 × PBS twice for 5 min and incubated with HRP-conjugated secondary antibody (Dako, Carpinteria, CA, USA) for 30 min, and HRP was detected by diaminobenzidine (DAB; Dako, Carpinteria, CA, USA). Tissue sections were counterstained with Mayer’s hematoxylin (Sigma, St. Louis, MO, USA) for 1 min at room temperature, incubated in 0.037M ammonia for 1 min, washed with distilled water for 5 min, and mounted with glycergel mounting medium (Dako, Carpinteria, CA, USA).

IHC images were captured using Pannormic Midi Digital Scanner (3DHISTECH Ltd., Budapest, Hungary) and viewed using CaseViewer program developed by 3DHISTECH (Budapest, Hungary). The analysis and quantification of IHC images were performed using ImageJ software (NIH, Bethesda, MD, USA). Each individual tissue sample on the TMAs was selected and converted to an 8-bit image, followed by measuring threshold. Two separate values were calculated. First, the region of total epithelial area (ROT) was defined and measured using ImageJ. Second, the total area with actual DAB staining signals in the defined epithelial structures was measured (region of interest, ROI). The relative mTOR staining intensities were determined based on the calculation of ROI/ROT × 100%. The statistical analysis was performed using ANOVA with Tukey’s post-hoc test for the multiple comparisons. The mTOR and AMACR antibodies were purchased from Cell Signaling Technology (Waltham, MA, USA) and Agilent Technologies (Santa Clara, CA, USA), respectively.

### 4.5. RT-qPCR Validation of miR-99b-5p

To measure the expression level of miR-99b-5p in various cell lines, RT-qPCR assay was performed. Total RNA was isolated using the miRNeasy Mini kit (Qiagen, Germantown, MD, USA) from each cell line. To quantify total RNA of samples, NanoVue Plus spectrophotometer (GE Healthcare, Wauwatosa, WI, USA) was used. Reverse transcription was performed using miRCURY LNA RT kit (Qiagen, Germantown, MD, USA), where 1 µg of total RNA was used as a template. Once cDNA was synthesized, quantitative PCR (qPCR) assay was performed using miRCURY LNA SYBR Green PCR kit (Qiagen, Germantown, MD, USA). The miRNA primers designed for amplification of hsa-miR-99b-5p and hsa-miR-103a-3p were based on the specific miRCURY miRNA Assays purchased from Qiagen (Germantown, MD, USA). The qPCR reaction program was set as follows: pre-denaturation for 5 min at 95 °C, followed by 40 standard cycles of: denaturation at 95 °C for 15 s, annealing at 55 °C for 30 s, and extension at 70 °C for 30 s. To determine the miRNA expression levels, qPCR reactions were performed in duplicates or triplicates from 3 independent RNA samples, using endogenous miR-103a-3p for data normalization. Normalized gene expression levels were determined using the 2^−ΔCT^ method as previously described [14,15].

### 4.6. Western Blot Analysis

The total proteins were extracted using M-PER extraction reagent with protease and phosphatase inhibitor cocktail (Thermo Fisher Scientific, Waltham, MA, USA) according to manufacturer’s protocol. Cytoplasm and nuclear proteins were extracted using Subcellular Protein Fractionation kit (Thermo Fisher Scientific, Waltham, MA, USA) according to the manufacturer’s protocol. Equal amounts of proteins were used based on the quantification using BCA assay kit (Thermo Fisher Scientific, Waltham, MA, USA), and the samples were separated by electrophoresis using NuPAGE 4–12% or 8% Bis-Tris gels (Invitrogen, Waltham, MA, USA). The gels were transferred to PVDF membranes (Bio-Rad, Hercules, CA, USA) then the PVDF membranes were incubated with SuperBlock blocking buffer (Thermo Fisher Scientific, Waltham, MA, USA). After 1h, the PVDF membranes were incubated with primary antibodies overnight at 4 °C, washed 3 times with 1 × TBST, and then incubated with secondary antibody for 1h at room temperature, then washed 5 times with 1 × TBST. The results were analyzed with SuperSignal ECL substrates (Thermo Fisher Scientific, Waltham, MA, USA) and a ChemiDoc XRS system (Bio-Rad, Hercules, CA). The primary and secondary antibodies used in the study were mTOR, pmTOR, AR, GAPDH, Lamin B1, β-actin, and anti-rabbit IgG-HRP antibodies from Cell Signaling Technology (Waltham, MA, USA).

### 4.7. Immunofluorescence Staining

In this process, 4 × 10^4^ cells were seeded on cover slip and allowed to adhere for 24 h in 5% CO_2_ incubator at 37 °C. All the cells were subjected to immunofluorescence assays 48 h following transfection. Briefly, cells were washed with 1 × PBS, fixed in 4% paraformaldehyde, and permeabilized with 0.1% Triton X-100. Cells were then blocked for 1 h with 2% BSA in 1 × PBS. Primary antibodies against mTOR (Cell Signaling Technology, Waltham, MA, USA) and pmTOR (Santa Cruz Biotechnology, Santa Cruz, CA, USA) were applied to the fixed/permeabilized cells for incubation overnight at 4 °C. The cells were washed twice with 1 × PBS, and followed by incubating with Alexa-Fluor-488-conjugated anti-rabbit and Alexa-Fluor-594-conjugated anti-mouse antibodies, respectively (catalog# A32731 and #A32744, from Invitrogen, Waltham, MA, USA) for 1 h at room temperature. Thereafter, cells were washed twice with 1 × PBS for 5 min and the nuclei were visualized by staining with DAPI from Invitrogen (catalog# P36981, Waltham, MA, USA). All labeled and/or prepared cells mounted on glass slides and were visualized by fluorescence microscopy (Olympus, Waltham, MA, USA). Cell images were captured from 3–4 random areas at 20× magnification by using CellSens V1.18 software (Olympus, Waltham, MA, USA).

### 4.8. TdT-Mediated dUTP-Biotin End-Labeling (TUNEL) Assays

TUNEL assay was carried out according to the manufacturer’s protocol (Click-iT™ Plus In-situ Apoptosis Detection with Alexa Fluor Dyes, Thermo Fisher Scientific, Waltham, MA, USA). Precisely, 4 × 10^4^ cells (HT-29, SW620, MDA MB 231, MCF-7, A549, and H1299) were seeded on coverslips and allowed to adhere for 24 h in 5% CO_2_ incubator at 37 °C. These cells were fixed in 4% paraformaldehyde in 1 × PBS after exposure of miR-99b-5p mimic or negative control mimic for 48 h, then washed with PBS three times followed by the incubation with 0.25% Triton X-100 in 1 × PBS and ultimately the cells were incubated with differential steps of TUNEL reaction mixture as per the manufacturer’s protocol. Lastly, the processed cells were counterstained with DAPI for 5 min, at room temperature in the dark. All labeled and/or prepared cells were mounted on glass slides and were visualized by Olympus BX3 fluorescence microscope (Olympus, Waltham, MA, USA). Cell images were captured from 3–4 random areas at 10× magnification by using CellSens V1.18 software (Olympus, Waltham, MA, USA).

### 4.9. Caspase 3/7 Activity-Based Apoptosis Assay

HT-29, SW620, MDA MB 231, MCF7, A549, and H1299 were seeded at a density of 3 × 10^4^ cells/well in 96-well plates. The cells were grown overnight and then followed by transfections. After 24 h, fresh media were applied to replace the transfection reagent-containing media with 11 mM of docetaxel or vehicle, then the cells were incubated for an additional 24 h. The Apo-ONE Caspase-3/7 Assay Kit (Promega Corporation, Madison, WI, USA) was used to measure apoptosis according to the protocol described by the manufacturer. Then, 100 µL of homogeneous Caspase-3/7 reagent was added to each well and the plate incubated at room temperature for 30min to 2h. Fluorescence was detected for measuring (at wavelengths of 499/521 nm excitation/emission) the apoptosis state (Caspase 3/7 activity) using Biotek Synergy HT Microplate Reader (BioTek, Winooski, VT, USA).

## Data Availability

Not applicable.

## Supplementary Materials

The following supporting information can be downloaded at: https://www.mdpi.com/article/10.3390/ijms23179643/s1.
