# Supplementary material for: Downregulation of miR-99b-5p and Upregulation of Nuclear mTOR Cooperatively Promotes the Tumor Aggressiveness and Drug Resistance in African American Prostate Cancer
Source: Int J Mol Sci. 2022 Aug 25;23(17):9643. doi: 10.3390/ijms23179643 (PMC9455949; doi:10.3390/ijms23179643)
Supplement: Supplementary file 1 [file ijms-23-09643-s001.zip › ijms-1879747-supplementary.pdf]

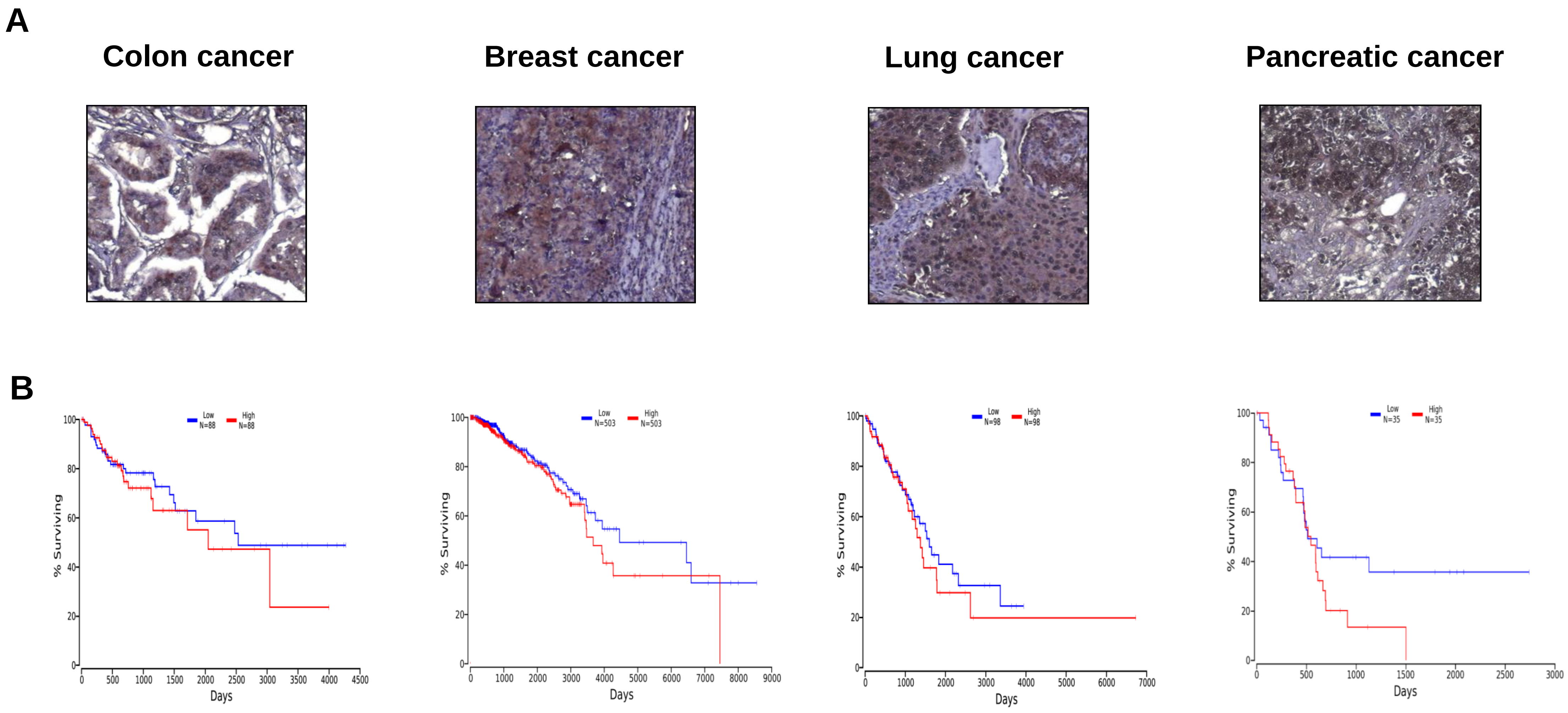

Supplementary Figure S1. (A) IHC staining of mTOR protein on a TMA containing colon, breast, lung, and pancreatic cancer specimens. The representative IHC images of mTOR staining from the indicated 4 cancer specimens the were presented. (B) Survival curves for cancer patients with high-level mTOR (red curve) and low-level mTOR (blue curve) expression levels. The mTOR expression data were obtained from TCGA-RNAseq database, and OncoLnc program (http://www.oncolnc.org/) was used to plot the survival curves.

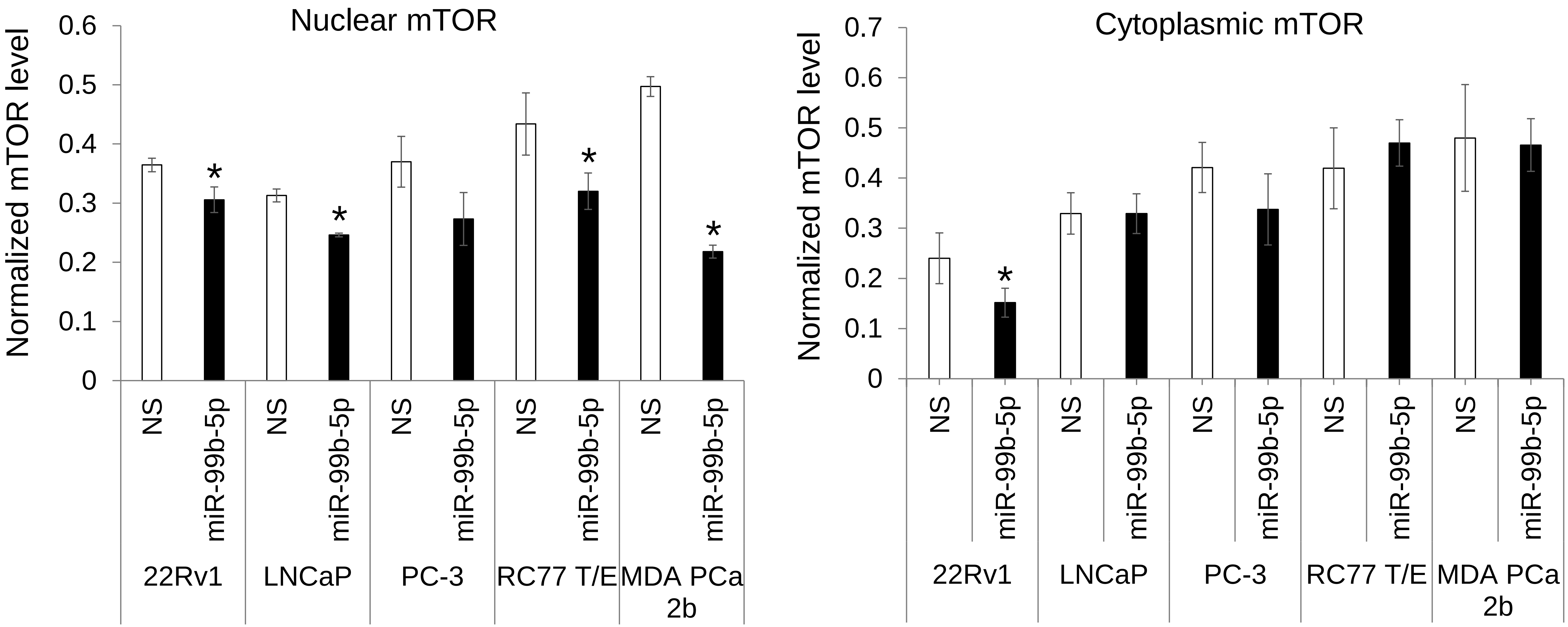

Supplementary Figure S2. Quantification of nuclear and cytoplasmic mTOR protein levels in PCa cells. The nuclear and cytoplasmic proteins extracted from the PCa cells (22Rv1, LNCaP, PC-3, RC77 T/E, and MDA PCa 2b) were subjects to the western blot analysis. Quantification of nuclear and cytoplasmic mTOR levels were determined by normalization of mTOR signals with Lamin B1 and GAPDH, respectively. Each value was represented as mean ± SD, based on 3-4 western blot images. Significant difference (*p*-value < 0.05, based on ANOVA with Tukey's post-hoc test) of normalized mTOR levels were shown between miR-99b-5p mimic vs. NS transfected PCa cells.

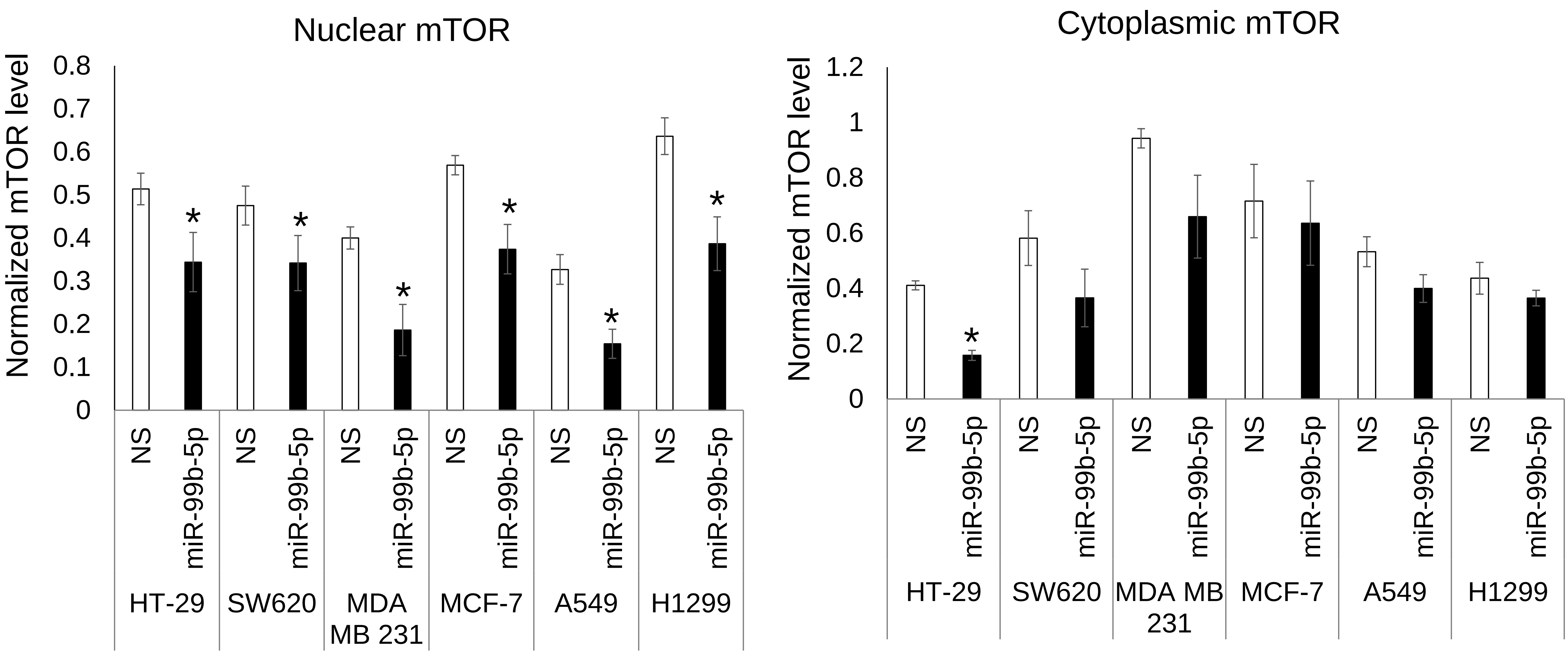

Supplementary Figure S3. Quantification of nuclear and cytoplasmic mTOR protein levels in colon, breast and lung cancers. The nuclear and cytoplasmic proteins extracted from the cancer cells (HT-27, SW620, MDA MB 231, MCF-7, A549, and H1299) were subjects to the western blot analysis. Quantification of nuclear and cytoplasmic mTOR levels were determined by normalization of mTOR signals with Lamin B1 and GAPDH signals, respectively. Each value was represented as mean ± SD, based on 3-4 western blot images. Significant difference (p-value < 0.05, based on ANOVA with Tukey's post-hoc test) of normalized mTOR levels were shown between miR-99b-5p mimic vs. NS transfected cancer cells.
